# Oral Cancer Risk and Screening Prevalence Among Hospitalized Women

**DOI:** 10.7759/cureus.61423

**Published:** 2024-05-31

**Authors:** Hussein Pothiwalla, Jerome Gnanaraj, Suchitra Paranji, Alexander Daniel, Waseem Khaliq

**Affiliations:** 1 Hospital Medicine, Johns Hopkins University School of Medicine, Baltimore, USA; 2 Hospital Medicine, Howard County General Hospital, Columbia, USA; 3 Otolaryngology-Head and Neck Surgery, Johns Hopkins University School of Medicine, Baltimore, USA

**Keywords:** prevalence study, prevalence, screening preference, hospitalized women, high-risk patients, oral cancer screening

## Abstract

Background

Oral cancer screenings are often on the back burner in the face of other cancer screenings. In high-risk individuals, early detection of oral cancer has a better prognosis and survival. Hospitalization may offer an opportunity to target high-risk populations. This study evaluates the prevalence of women at high risk for oral cancer among hospitalized women and their preference for oral cancer screening.

Design and participants

Five hundred and ten cancer-free women admitted to the hospital under the internal medicine service at an academic center were enrolled to participate in the study. Three hundred and seventy women were at high risk for developing oral cancer, defined by smoking status, alcohol use, or both. High-risk women received bedside smoking cessation counseling and oral cancer informational handouts and were offered oral screening examinations during hospitalization. Six months after discharge, study participants received a follow-up phone call to determine if these women discussed oral cancer screening with their primary care physicians at the follow-up visit.

Results

Seventy-three percent of the hospitalized women were at high risk for developing oral cancer. Fifty-seven percent of high-risk women reported having no primary dentist. High-risk women were more likely to be younger, reported a disability, and had a lower comorbidity burden than the average-risk group. Only 41% of high-risk hospitalized women received oral cancer screening examinations during the hospital stay. Post-hospitalization, 66% of high-risk patients discussed oral cancer screening with their primary care.

Conclusion

Almost three-fourths of hospitalized women are at high risk for developing oral cancer. Hospitalization provides an opportunity to educate and screen high-risk populations.

## Introduction

Oral cavity cancer is a type of head and neck cancer. An estimated 58,450 adults in the United States will be diagnosed and 12,230 will die with oral or oropharyngeal cancer in 2024 [1]. Ninety percent of all cases of oral cavity and pharyngeal cancer are classified as squamous cell carcinoma. At the time of diagnosis, more than 50% of patients with oral and pharyngeal cancer have regional or distant metastases [2]. The five-year survival rate among women is 86% for localized disease compared to 39% for those with distant metastasis [1]. Across the globe, oral cancers cause a significant financial burden, costing anywhere between 18% and 215% of the GDP per capita in some Western countries [3,4]. Head and neck cancer represents a heterogeneous group of malignancies, and treatment is often multimodal, consisting of varying combinations of surgery, radiation therapy, and systemic therapy. Oral cancer accounts for 3% of all cancer diagnoses and approximately 3% of all cancer treatment costs. In 2015, oral cancer treatment costs were $5.1 billion (including medical services and oral prescription drug costs), projected to be $6.7 billion by 2030 (~30% increase) [5]. Thus, screening for oral cancer may help identify early cancerous lesions that can be successfully treated.

The United States Preventive Services Task Force (USPSTF) reports insufficient evidence to recommend oral cancer screening; however, screening by visual examination has been shown to reduce mortality rates in high-risk populations [6,7]. An opportunistic oral cancer screening conducted in primary care or dental office settings for high-risk patients is feasible, cost-effective, and not resource-intensive [8]. The standardized oral examination recommended by the National Institute of Dental and Craniofacial Research is efficient and typically lasts only a few minutes [9]. Risk factors for oral cancer include alcohol, tobacco, and sexually transmitted human papillomavirus (HPV) infection. In the United States, approximately 65% of oral and pharyngeal cancer cases in women are attributable to smoking and alcohol use [10].

Preventive care screening utilization is strongly associated with having a primary care physician visit in the past year [11]. Studies suggest that hospitalized patients reflect upon their health in general during an inpatient stay and might be more receptive to preventive care interventions [12,13]. Although hospitalization is a critical illness event, it can provide downtime for preventive health interventions like education, counseling, and screening to a captive high-risk audience. Thus, screening during hospitalization can help overcome barriers associated with screening, like transportation, convenient scheduling of appointments, and time off from work.

The primary objectives of our study were threefold: evaluate the prevalence of hospitalized women at high risk for developing oral cancer, their cancer screening attitudes towards other common cancers (breast and colorectal), and their willingness to undergo oral cancer screening during hospitalization. Secondary outcomes were to determine the feasibility of a brief oral cancer screening during hospitalization and ascertain if these women discussed oral cancer screening with their primary care providers at follow-up visits post-hospitalization. The reason for selectively including women is that women tend to prioritize breast and cervical cancer screenings and do not perceive themselves as at risk for developing other cancers like colorectal and lung [14,15].

## Materials and methods

The current study is an observational prospective study; a detailed enrollment method has been described in a prior publication [14]. We prospectively enrolled 510 women aged 50-75 years who were cancer-free at baseline (excluding non-melanoma skin cancers) and admitted to the medicine service at Johns Hopkins Bayview Medical Center between December 1, 2014, and May 31, 2017. Patients were excluded from the study if their life expectancy was less than 10 years like dementia, end-stage liver disease, acquired immunodeficiency syndrome, hospice care, and end-stage renal disease on hemodialysis. Patients were also excluded if current hospitalization was for acute mental status change, acute myocardial infarction, acute stroke, acute pulmonary embolism, pregnancy, and acute fulminant hepatitis. Patients with multiple admissions during the study period were offered enrollment on their first hospital admission. Women who were non-smokers and reported no alcohol use were categorized as average risk, whereas women who reported smoking, alcohol use, or both smoking and alcohol use were categorized as high risk. Oral cancers that are amenable to screening using a visual and manual examination of the oral cavity and oral pharynx were the focus of the current study.

Protocol and measures

The study coordinator utilized a bedside survey to collect data on socio-demographics and health behavior such as smoking status, alcohol use, and screening practices for breast cancer (BRC) and colorectal cancer (CRC). Hospitalized women were categorized as high risk if they were current or ex-smokers, alcohol users, or both. In contrast, average risk was defined as women who were non-smokers and denied current alcohol use. Access to healthcare was assessed by health insurance status and having a primary care physician. Disease burden was evaluated by assessing medical comorbidities, including those needed for the Charlson Comorbidity Index (CCI), which categorizes comorbidities by mortality risk status based on the International Classification of Diseases (ICD) diagnosis codes [16].

Study intervention

The intervention consisted of bedside counseling about smoking cessation and an oral cancer informational handout for women in the high-risk group (see Appendices for intervention handout). High-risk women were asked if they would be amenable to having a brief oral cancer screening (examining the oral cavity with a penlight and jaw/neck external palpation for lymph nodes and salivary gland exam) during the current hospitalization. Once a patient agreed to have an oral screening examination, the primary hospital provider was made aware of the patient's willingness to screen. All study participants received a $10 gift card at the completion of study enrollment. A penlight was provided to all hospital providers free of cost (physicians, physician assistants, and nurse practitioners) prior to starting the study to encourage oral cavity examination for patients amenable to oral cancer screening during hospitalization. Inpatient providers' hospital documentation was ascertained post-intervention to confirm oral cancer screening examination. Six months after discharge from the hospital, study participants started to receive a follow-up phone survey to elicit if patients had followed up with their primary care provider and discussed oral cancer screening post-hospitalization with their primary care provider. Up to three attempts were made to reach the study participants for follow-up phone surveys.

Outcome and evaluation

The primary outcome was to evaluate the prevalence of women at high risk for developing oral cancer, their attitude towards other common cancers (BRC and CRC), and their preference for oral cancer screening during hospitalization. The secondary outcome was determining the feasibility and frequency of high-risk hospitalized women undergoing brief oral cancer screening during hospital stay.

Statistical methods

Respondent characteristics are presented as proportions and means. Unpaired t-tests and chi-squared tests were utilized to compare the demographic and socioeconomic characteristics of hospitalized women at high risk versus average risk for oral cancer. T-tests and chi-squared tests determined significance at a p-value ≤0.05. The study sample size was determined using a 5% margin of error with a confidence level of 95% and a response distribution of 50%. The data were analyzed using the Stata Statistical Software: Release 13.1 (2013; StataCorp LLC, College Station, Texas, United States). The Institutional Review Board/Ethics Committee of Johns Hopkins Bayview Medical Center approved the study protocol (study approval number: IRB00049608). All study participants provided their written informed consent for participation.

## Results

Of these 510 women, 140 women were at average risk (non-smokers and reported no alcohol use), whereas 370 women were at high risk (smoking only (n=238), alcohol use only (n=49), and both smoking and alcohol use (n=83)). Almost three-fourths (73%) of the study population was at high risk for developing oral cancer, the mean age was 60.5 years (SD=6.9), 45% reported an annual household income of less than $20,000, and 36% were African American. Detailed study population characteristics stratified by risk for oral cancer can be viewed in Table 1.

**Table 1:** Characteristics of the study population by oral cancer risk stratification ◊For some patients, the variables had a missing value *Chi-squared, Fisher's exact statistic (where at least 20% of frequencies were <5), and unpaired t-test statistic ¥CCI: scores of 0, 1, 2, and 3 predicting 10-year survival rates of 93%, 73%, 52%, and 45%, respectively CCI: Charlson Comorbidity Index

Characteristics^◊^	Average risk (n=140)	High risk (n=370)	P-value^*^
Age in years, mean (SD)	62.1 (7.1)	59.9 (6.8)	<0.001
Race			0.18
Caucasians, n (%)	78 (56)	231 (62)	-
African American, n (%)	59 (42)	125 (34)	-
Others, n (%)	3 (2)	14 (4)	-
Married or living with a partner, n (%)	43 (31)	112 (30)	0.92
High school or more years of education, n (%)	114 (81)	292 (79)	0.53
Employment status, n (%)			0.02
Employed	25 (18)	88 (24)	-
Unemployed	9 (6)	25 (7)	-
Retired	53 (38)	88 (24)	-
Disability/unable to work	53 (38)	169 (46)	-
Chronic disabled, wheelchair-bound, or bed-bound, n (%)	9 (6)	16 (4)	0.60
Annual household income	70 (50)	155 (43)	0.11
Uninsured, n (%)	0 (0)	2 (1)	0.25
No primary care physician, n (%)	9 (6)	37 (10)	0.23
Admitted as observation, n (%)	10 (7)	19 (5)	0.4
Principal diagnosis at admission, n (%)			0.92
General internal medicine, n (%)	48 (34)	113 (31)	-
Cardiovascular, n (%)	20 (14)	58 (16)	-
Pulmonary, n (%)	22 (16)	72 (20)	-
Gastrointestinal, n (%)	18 (13)	47 (13)	-
Neurology, n (%)	4 (3)	3 (1)	-
Nephrology, n (%)	10 (7)	24 (6)	-
Oncology, n (%)	2 (1)	6 (2)	-
Rheumatology, n (%)	5 (4)	13 (4)	-
Psychiatry, n (%)	1 (1)	3 (1)	-
Infectious disease, n (%)	6 (4)	19 (5)	-
Others, n (%)	4 (3)	12 (3)	-
Length of stay in days, mean (SD)	5.4 (7.9)	4.8 (3.6)	0.24
Mean BMI kg/m^2^, (SD)	34.9 (12.6)	32.6 (9.5)	0.03
Age-adjusted CCI,^¥^ mean (SD)	3.7 (1.9)	3.2 (1.7)	0.007

The population characteristics varied between these two groups; for example, high-risk women were more likely to be from the younger age group and reported chronic disability, whereas average-risk women were more likely to be retired, had obesity, and carried a higher comorbidity burden when compared to high-risk women. We found no difference between the two groups regarding health behavior like cancer screening (oral cancer screening, screening colonoscopy, or screening mammography). Fifty percent of hospitalized women at high risk for developing oral cancer never had an oral cancer screening. Hospitalized women at average risk and high risk were equally non-adherent to BRC and CRC screening, as noted in Table 2.

**Table 2:** Care received during hospital admission and preferences for screening while hospitalized ŦFor some patients, the variables had a missing value *Chi-squared and Fisher's exact statistic (where at least 20% of frequencies were <5)

Questions^Ŧ^	Average risk (n=140)	High risk (n=370)	P-value^*^
Never had oral cancer screening, n (%)	59 (43)	180 (50)	0.18
Never had screening colonoscopy, n (%)	46 (33)	127 (34)	0.76
Never had screening mammography, n (%)	11 (8)	28 (8)	1.00
Non-adherent to colorectal cancer screening, n (%)	33 (24)	104 (28)	0.30
Non-adherent to screening mammography, n (%)	41 (29)	128 (35)	0.23
No primary dentist, n (%)	67 (48)	211 (57)	0.08
How frequently do you visit your dentist?			0.44
Every six months, n (%)	39 (55)	70 (45)	-
Once a year, n (%)	17 (24)	40 (26)	-
Only visit as needed, n (%)	15 (21)	46 (29)	-
Never, n (%)	0 (0)	1 (0)	-
Study intervention: bedside counseling about smoking cessation and handout about oral cancer	-		-
Willing to have oral cancer screening during hospital stay, n (%)	-	176 (48)	-
During a hospitalization, received oral cancer screening, n (%)	-	152 (86)	-

Access to primary dentists was similar between the two groups by asking if they have a primary dentist for dental care. We found that 48% (n=176) of hospitalized women at high risk for oral cancer were willing to undergo oral cancer screening during hospitalization, of whom 86% (n=152) received oral screening during hospital stay. Fifty-seven percent of hospitalized women at high risk for oral cancer reported no primary dentist as compared to 48% at average risk. There were no abnormalities or precancerous lesions reported during the oral screening examinations during the study period. 

We could only reach 123 women (24%) of the study population post-hospitalization, two-thirds of whom were at high risk (n=95). Among high-risk group women, 97% (n=88) reported following up with their primary care provider, and 66% (n=57) reported discussing oral cancer screening with their primary care provider. There was no difference in follow-up with primary care provider and discussion about oral cancer screen with primary care provider at post-discharge follow-up between the two groups.

## Discussion

Our study showed that over three-quarters of the women hospitalized were at high risk of developing oral cancer. There is no significant difference in the screening behavior between the high-risk and average-risk groups, as they were both equally non-adherent to other cancer screenings. The study also showed that almost half of women in the high-risk group are willing to undergo oral cancer screening during hospitalization. These findings from our study are significant as they show not only the willingness but also proof of the feasibility of oral cancer screening during a hospital stay, especially for patients at high risk for oral cancer. Such screening might be helpful to potentially capture some of the oral cancer patients at the early stages. The study also reported that almost two-thirds of women from the high-risk group discussed oral cancer screening with their primary care providers at follow-up visits post-hospitalization.

The stage of cancer at diagnosis is the single most important determinant in the prognosis. Thus, early detection of cancers is the key to reducing morbidity and mortality as well as the financial burden of the disease. Individuals with oral cancers are expected to be particularly vulnerable to financial strains, given the established association with lower socioeconomic status [17]. The average cost of oral cancer in the first year after diagnosis was $79,151, significantly higher than the cost to treat other cancers ($31,559-65,123) [4]. This trend continues even after the first year, where the cost incurred for medical care is twice as much as any other reported cancer. The higher cost is attributed to the multiple modalities of treatment involved and the number of late-stage diagnoses [4,18]. Approximately 88% of oral cancers are detected in the Medicare-eligible population, that is, over 65 years of age [19]. However, Medicare covers only certain medically necessary dental procedures [20], making the Medicare population pay out of pocket for some or most of their dental care which might be a barrier to seeking preventative dental healthcare. Therefore, providing a quick, low-risk screening test while they are hospitalized might mitigate some of the downstream effects of lack of dental care access.

The current guidelines set forth by the USPSTF found inadequate evidence that screening for oral cancer and treatment of screen-detected oral cancer improves morbidity or mortality. However, the guideline also noted a lack of evidence on the harms of screening, stating that the natural history of screen-detected oral cancer or potentially malignant disorders is unclear [7]. This recommendation differs from the American Cancer Society, which recommends that adults aged 20 years or older who have periodic health examinations should have the oral cavity examined as part of a cancer-related checkup [21], and the American Dental Association, which recommends clinicians perform visual and tactile oral examination in all adult patients [22]. Population-based studies have demonstrated that a primary care screening strategy for the early detection of head and neck cancer reduces overall mortality in the general population and can reach many at-risk individuals [23].

Our study identified that a vast majority of the hospitalized women were at high risk for oral cancer; furthermore, a significant portion of these women were African Americans or from the low-income group where barriers to healthcare access and healthcare disparities exist. Hospitalizations can be life-altering events where patients reflect upon their overall health and are generally more receptive to recommendations made by their hospital providers. Thus, opportunities to provide simple healthcare screening to capture the group, especially at high risk and vulnerable, should be considered part of comprehensive inpatient care.

Several limitations of this study should be considered. First, the study was conducted at a single hospital. Second, HPV is a significant risk factor for oropharyngeal cancers, but our selection criteria did not ascertain prior HPV infection. Third, we did not solicit input about the potential impact of counseling and screening from the healthcare organization and hospital provider, especially given the time constraints in an acute care setting. Fourth, barriers like assigning low priority to cancer screening might exist in the face of a more serious health issue during acute hospitalization. Fifth, only women were enrolled in this study; however, women perceive risk for cancers differently than BRC and CRC. For example, gender disparity persists in CRC screening utilization [24,25]. Thus, this can be considered the study's strength. Finally, recall bias might also exist since the bedside survey answers are self-reported. Nevertheless, we found that many high-risk hospitalized women were open to undergoing oral cancer screening while in the hospital. Further, they were willing to discuss oral cancer screening with their primary care doctor.

Dental care in the United States is still subject to healthcare disparities, and oral healthcare utilization for US adults has declined during the past decade especially among the poor and uninsured [26]. In many states, the gap in rates of oral healthcare between disadvantaged and advantaged adults has been widening and may have already gotten worse with the COVID-19 pandemic [26]. For such populations, providing inpatient dental screening can be an ideal opportunity to uncover a premalignant lesion before it leads to morbidity and intense resource utilization in the future.

Given the era of automatic electronic health records (EHR) alerts, incorporating risk stratification tools in the EHR and alerting the healthcare provider of high-risk individuals might prompt a discussion about oral cancer screening. Time permitting the performance of an oral examination might identify some precancerous lesions. We must work out feasibility issues for oral cancer screening as our healthcare systems move towards patient-centered care, regardless of clinical locale. This is especially necessary for high-risk and vulnerable populations that will also require a close follow-up care plan if cancer or precancerous lesions are detected.

## Conclusions

The prevalence of risk factors for oral cancer is high among hospitalized women. Hospitalization provides an opportunity to identify and counsel these patients, who reflect upon their health and are receptive to recommendations by their hospital providers to undergo oral cancer screening. Although the study provides evidence for the feasibility of inpatient oral cancer screening, future studies are needed to evaluate the feasibility of chair-side screening examination, cost-effectiveness, and careful post-hospitalization follow-up plans for patients with suspicious oral lesions.

## Figures and Tables

**Figure 1:**
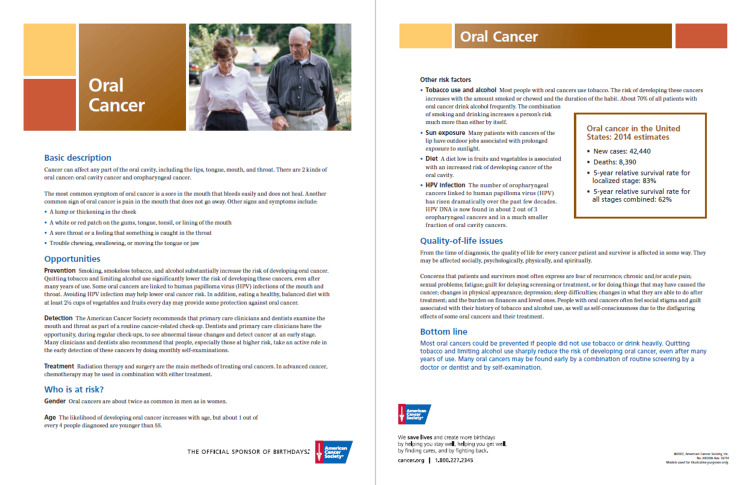
Intervention utilized for the study
